# Mathematical Psychiatry: On Cortical Spreading Depression—A Review

**DOI:** 10.3390/brainsci13091241

**Published:** 2023-08-25

**Authors:** Ejay Nsugbe

**Affiliations:** Nsugbe Research Labs, Swindon SN1 3LG, UK; ennsugbe@yahoo.com

**Keywords:** mathematical psychiatry, cortical spreading depression, migraine with aura, mathematical medicine, migraines, epidemic, mathematical psychology

## Abstract

The concept of migraine with aura (MwA) is a widespread condition that can affect up to 30% of migraine patients and manifests itself as a temporary visual illusion followed by a prolonged headache. It was initially pitched as a neurological disease, and observed that the spread of accompanying electrophysiological waves as part of the condition, which came to be known as cortical spreading depression (CSD). A strong theoretical basis for a link between MwA and CSD has eventually led to knowledge of the dynamics between the pair. In addition to experiment-based observations, mathematical models make an important contribution towards a numerical means of expressing codependent neural-scale manifestations. This provides alternate means of understanding and observing the phenomena while helping to visualize the links between the variables and their magnitude in contributing towards the emanation and dynamic pulsing of the condition. A number of biophysical mechanisms are believed to contribute to the MwA-CSD, spanning ion diffusion, ionic currents of membranes, osmosis, spatial buffering, neurotransmission, gap junctions, metabolic pumping, and synapse connections. As part of this review study, the various mathematical models for the description of the condition are expressed, reviewed, and contrasted, all of which vary in their depth, perspective, and level of information presented. Subsequent to this, the review looked into links between electrophysiological data-driven manifestations from measurements such as EEG and fMRI. While concluding remarks forged a structured pathway in the area on sub-themes that need to be investigated in order to strengthen and robustify the existing models, they include an accounting for inter-personal variability in models, sex and hormonal factors, and age groups, i.e., pediatrics vs. adults.

## 1. Introduction

Migraines are widespread debilitating disorders that have been described by the World Health Organization (WHO) alongside a list of other headache-related conditions [1,2,3]. Migraines can be characterized as an epidemic that is estimated to affect 14% of the world’s population and is three times more common in women than in men [1,2,3]. Among migraine sufferers, MwA has been said to occur around 30% of the time and is accompanied and characterized by illusions in the visual field that are created within the visual cortex [4]. The cerebral manifestation of the condition was uncovered while work was being carried out to interpret cortex-based electrogram readings during epileptic seizures in rabbits [4]. It was noted that during epileptic seizures, sporadic epileptic waves are succeeded by slow waves that inhibit the overall excitability within the cortex of the brain, which has been termed “cortical spreading depression”, or CSD [5]. This phenomenon was further solidified with a data-driven link between CSD and MwA, where blood oxygen levels were measured using functional MRI and displayed apparent characteristics of the CSD [4,5]. Despite the fact that an abundance of data has been collected in the area of neurophysiology, outlining the causal effects of MwA has proven to be a challenge due to the highly dynamic nature of the event itself [5].

The concept of MwA is assumed to be connected with spreading waves of hyperexcitations in the form of spreading depolarizations in the brain, which are quickly followed by reduced blood flow and a suppression of overall neural activity and are characterized by spreading depression [6,7,8,9]. It can be noted that the occurrence of spreading depolarizations and cortical spreading depressions provides a phenomenological abstraction of the process of MwA [6,7,8,9]. The contributions presented in this work aim to first survey and synthesize the various models that quantitatively describe the CSD and MwA phenomena, alongside recommendations for how the subsequent models in this area can be assembled to be more comprehensive and account for more aspects of the phenomena. Predominantly, robust recordings of electrophysiological manifestations of the CSD in action have been challenging to acquire due to the intermittent nature of the condition; plus, there would be the need for invasive cortical recordings to capture the dynamics of the ion channels, etc., during the event.

Explicitly speaking, the aim of this review is to achieve the following:

- To survey and synthesize the mathematical psychiatric models adopted towards accounting for CSD while highlighting the strengths of each;
- To discuss the notion and pathway for improvements where further work can be carried out to robustify the models existing in the literature;
- To flesh out a number of uncertainties that require further investigations in order to foster meaningful advancement in the area of research.

As part of this work, mathematical models on the topic are employed in the quantitative descriptions and modeling of the CSD [10]. Mathematical models have proven to be insightful and invaluable over the years for the identification, modeling, and provision of controlled and safe “what-if” behavior analyses, along with overall support towards the understanding of biological and medical system functions [10,11].

## 2. Concept of Migraine-Induced Auras and Hallucinations

Auras and hallucinations manifest when sensory events occur without any external trigger stimuli. These migraine auras usually manifest themselves prior to the headache itself by around 10 min [12,13,14,15,16,17,18,19,20]. Aura hallucinations have been said to be unilateral, occasionally bilateral, and even on the same side as the headache [12,13,14,15,16,17,18,19,20]. Common symptoms of the MwA include blurry vision, seeing flickering lights, and some cases of myopia [12,13,14,15,16,17,18,19,20]. The more common modes of hallucinations for migraines are the fortification spectrums, which have been seen to be a key feature in distinguishing and identifying migraines from various alternate forms of disorders [12,13,14,15,16,17,18,19,20]. These apparent phenomenologically-based manifestations have formed the characteristics that have been incorporated into mathematical models of the MwA and subsequent CSD, as reviewed in Section 8 of this paper.

## 3. Physiological Aspects of MwAs

**Figure 1:**
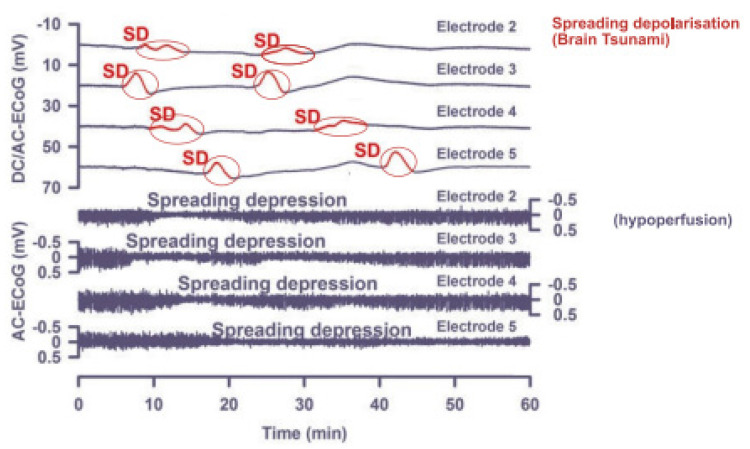
Electrocorticography measurements showing a sequence of depolarization and subsequent suppression. The top plot shows direct current (DC) activity, while the bottom plot shows the alternating current (AC) equivalent [9].

CSDs are largely characterized by a series of “self-propagating” streams of neural wave activities that have been described as “brain tsunamis”, and are succeeded by suppression and depressions across the cortex [12,13]. A series of snapshots showing the sequence of depolarization and subsequent suppression acts from electrocorticography (EcoG) measurements can be seen in Figure 1. The measurements were acquired from a chronically ill patient who suffered a stroke.

Subsequent to the initial wave of depolarization, a hyperpolarization act occurs, which involves the hyperpolarization of neurons and a reversal of the membrane polarity, which ensures that there is no further generation of synaptic potentials [9]. As the suppression activity occurs and the overall excitability is reduced, the CSD parallels the wave of depolarization, where the recovery process can typically span minutes and up to an hour [9].

**Figure 2:**
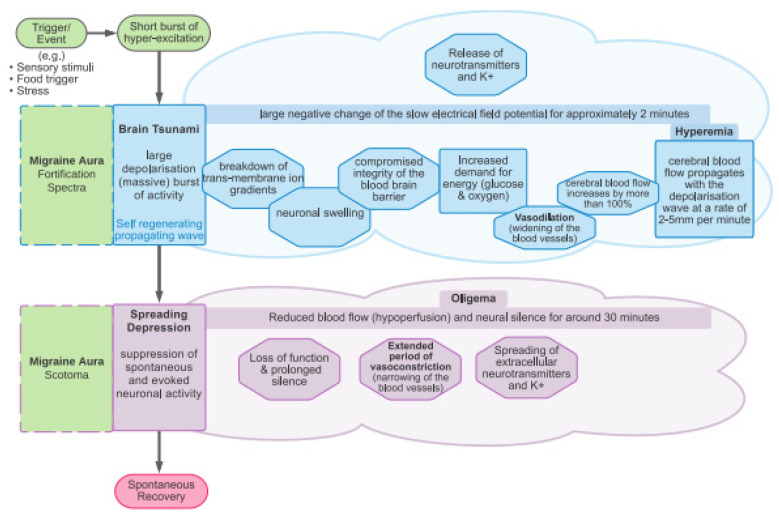
Annotated sequence of the CSD phenomenon showing simultaneous spreading depression and migraine scotoma, along with the negative potential shift of the depolarization referred to as the brain tsunami [9].

Figure 2 showcases annotations related to the CSD phenomenon, from which it can be seen that the spreading depression and the migraine scotoma commence simultaneously, alongside the negative potential shift of the depolarization referred to as the brain tsunami.

Pioneering investigators in the area of CSD, such as Leão et al. [16], observed saturation in spontaneous neural activity once electrical stimulation was applied to animals such as rabbits and pigeons. However, this is challenging in the case of human beings due to the need for subdural electrodes, which mostly tend to be utilized after traumatic brain injury [9]. Noninvasive EEG recordings have, as yet, not been shown to have captured the spreading depolarization effects due to their low spatial resolution [9]. Thus, CSD has been mostly investigated in animals and then used as a basis for a “scale up” in humans, allowing for closer investigations of the factors influencing altered cortical and subcortical excitations and inhibitions [9]. This has provided useful starting points for the mathematical models that have been formulated to further describe the phenomenon [9].

## 4. CSD

The manifestation of CSD involves the steady streaming and pulsing of chemical waves, which have the ability to reduce excitability of tissues within the neurons and ultimately lead to changes to both intracellular and extracellular ionic concentrations, i.e., sodium and potassium [4,21,22]. Sequences of CSD waves can be generated using stimuli such as potassium chloride on the cortical surface, mechanical impulses, and targeted electrical stimulation mechanisms. From a dimensional perspective, in 2D/3D, CSD waves propagate through layers of gray matter, while in 1D approximation, the behavior of these waves has properties that can be likened to the propagation of action potentials of nerve axons [4,21,22]. Properties CSD waves include solitary and refractory behaviors, while a potential “head-on” collision between a pair of CSD waves would lead to both waves canceling each other out [4,21,22]. The key distinguishing properties between CSDs and typical action potentials include the notion that the dynamic behaviors of CSDs are on a much larger scale, i.e., CSD evolution is measured in minutes (mm/min), while action potentials occur over milliseconds (m/ms) [4,21,22].

Due to the qualitative complexity of CSD, mathematical models have received emphasis for their use in studying the phenomenon, with a focus on various aspects and mechanisms related to it [4,21,22]. As always, the study of brain-related phenomena allows for a better understanding of how the brain functions as a whole, while the associated mathematical models particularly assist in the understanding of granular phenomena in the brain related to CSD, which would have been challenging to observe and isolate due to the multifactorial nature of the manifestation [4,21,22]. Specifically, the case study of CSD provides neuroscientific knowledge to use as a tool for learning about the brain. Moreover, the use of mathematical models can allow for the study of complex systems alongside their properties, while the use of these theoretical models enables researchers to systematically hold/control various segments of the interactions to see how the part influences the whole, a feat that may not be feasible or ethically acceptable in real-life studies [4].

## 5. CSD Mathematics

As mentioned, a precise underpinning of the manifestation of CSD continues to be challenging, despite the experimental observations where mathematical tools have been employed in efforts to contribute towards a clearer understanding of the phenomenon [4]. Amongst the mathematical literature, the use of discrete and continuum-based modeling methods can be utilized towards describing the CSD mechanisms, while the deeper literature in the area has shown attempts towards modeling CSD phenomena using cellular automata or Hodgkin-Huxley-based neuronal models [4].

The employment of discrete and continuum-based models in tandem allows for the representation of neurons as specific and discrete models that provide electrochemical stimuli, while the use of continuum-based models also allows for temporal evolution and spatial dependencies with the use of ordinary differential equations (ODEs) and partial differential equations (PDEs) [4]. These models are developed with the assumption of mean behaviors over intracellular spaces (ICS) and extracellular spaces (ECS) of both the neurons and glia, with each of the extracellular and intracellular spaces treated as a continuum space [4].

## 6. Components of the CSD

The majority of mathematical models are assembled and derived under specific assumptions and reduced operating conditions, and as part of the CSD modeling, it can be seen that mostly key and essential components of the interactions are accounted for and included [23,24]. The key components typically considered as part of the CSD models in the literature are described as follows and comprise a mix of physical and physiological components:

### 6.1. Ion Diffusions

Ion diffusions occur as a result of the redistribution that occurs in the extracellular and intracellular components and thus need to be accounted for as part of the modeling process. Grafstein et al. stressed the importance of extracellular potassium and its relevance to CSD, where the ion diffusions are fairly constrained due to the presence of cells and the vascular networks [4,25]. From the perspective of a CSD phenomenon, the diffusion act can be pseudo-characterized using the medical tortuosity concept, i.e., mostly a non-linear propagation dynamic, which can be accounted for with existing mathematical models [4,25]. The lattice Boltzmann method has been previously used to account for tortuosity in simulations from the viewpoint of a diffuse substance permeating and propagating through cellular structures [4,25]. For brain-cellular conditions, related work has assumed times to be in the region of 1.6, whereby the 1.6 reflects the fact that the pace of the diffusive substance is damped by a relevant factor of 1.6 [4,25].

On the dimensions of length, it can be said that the diffusion of the ICS in the neurons can be approximated as negligible, whereas the effect of diffusions is more considerable within the glial cells, as the glial networks are linked with syncytial nets that allow for ions to travel through the networks [4]. As a way of accounting for the dynamic behavior of intracellular ion transport in the glia syncytium, a diffusion coefficient is considered, as inspired by the work of Chen and Nicholson [26].

### 6.2. Membrane Channels and Pumps

Due to the dynamics of the CSD, membrane behavior and currents behave differently than under standard conditions. In addition to this, not all channels are active during the depolarization of the membrane potential; hence, the identification of these explicit channels is somewhat challenging [4]. Furthermore, there are membrane channels that consume energy and move ions oppositely to their electrodiffusive rhythms. These channels expend a substantial amount of energy and are required to reach brain homeostasis equilibrium. A descriptive list of the channels and pumps can be seen as follows:

#### 6.2.1. Ion Channels

Prior work has been conducted on the identification of the particular channels involved in the spreading of the CSD phenomenon [4,21]. For example, despite the fact that fast sodium channels are key to generating neuronal action potentials, they are simply not pivotal in the spread of CSD, as the inhibition of these pathways and channels does not deter the CSD [4,21]. In contrast, the N-methyl-d-aspartate (NMDA) pathways have been seen to make a critical contribution towards the manifestation of the CSD, and the blocking of this pathway can block and inhibit its spread [4,21]. A mathematical representation showing the channel currents for each ion is the Hodgkin–Huxley model, as seen in Equation (1):

(1) $$I_{ion.HH}=g_{ion.HH}\left(E_{m}-E_{ion}\right)$$

where $g_{ion.HH}$ represents the conductance for each of the ions, and $E_{m}$ and $E_{ion}$ are the membrane and Nernst potentials for each ion. However, a more insightful model that amalgamates both theoretical postulations and experimental observations reflects that channels can both be activated and deactivated depending on the ionic current of the ECS and ICS, as indicated by the Goldman-Hodgkin-Katz (GHK) model in Equation (2):

(2) $$I_{ion.GHK}=m_{ion}^{p}n_{ion}^{q}\frac{FP_{ion.GHK}E_{m}\left({\left[ion\right]}_{i}-\exp\left(-\frac{E_{m}}{\emptyset }\right){\left[ion\right]}_{e}\right)}{\emptyset \left(1-\exp\left(-\frac{E_{m}}{\emptyset }\right)\right)}$$

where $m_{ion}\;and\;\;n_{ion}$ represent the activation and inhibitory factors of the gatings, p and q are integers with respect to the channel type, F is the Faraday constant, $\emptyset =\frac{RT}{F}$, while $P_{ion.GHK}$ represents the permeability. In this equation, R and T stand for the universal gas constant and absolute temperature, respectively, while ${\left[ion\right]}_{i}$ and ${\left[ion\right]}_{e}$ are the intra and extracellular concentrations, respectively. A more granular description can be seen in the work conducted by Bennett et al. [27].

#### 6.2.2. Ion Pumps

A variety of sodium pumps exist, including the potassium-sodium interchange pump, which replaces two potassium-sodium ions for every three removed from the cell and is seen to be among the most important [4,27]. The mathematical representation of this is via the Hill-type dependency on extracellular potassium and intracellular sodium, expressed as Equation (3):

(3) $$I_{k.pump}=-rN_{ak}{\left(\frac{{\left[k\right]}_{e}}{{\left[k\right]}_{e}+\alpha_{e}}\right)}^{2}{\left(\frac{{\left[Na\right]}_{i}}{{\left[Na\right]}_{i}+\alpha_{i}}\right)}^{3}f\left(E_{m}\right)\;I_{Na.pump}=-\frac{3}{2}I_{k.pump}$$

where ${\left[k\right]}_{e}$ and ${\left[Na\right]}_{i}$ represent the extracellular potassium and intracellular sodium concentrations, $rN_{ak}$, $\alpha_{e}$, and $\alpha_{i}$ are constants, and $f\left(E_{m}\right)$ is a direct function of the membrane potential $E_{m}$. The values of these can be obtained via the use of iterative solvers.

#### 6.2.3. Spatial Buffering

The concept of spatial buffering stems from the acts of biophysical properties, where in a glial syncytium the cluster of cell connections stay as an iso-potential source [4,28]. An increment in the extracellular concentration of potassium results in a depolarization effect in the glial cells, which ultimately spreads to further regions within the syncytium [4,28]. An unbalance in the spatial formation of the membrane potential difference gives rise to local currents, as the membranes of glial cells show a high level of conductance towards potassium [4,28]. The potential difference results in a flow of sodium at the location of the depolarization subsequent to potassium in the ECS at more distant areas where its magnitude is still at a low level [4,28]. This potassium is then transported via a diffusion process to regions of lower concentrations in an efficient fashion. The current loop circuitry is closed off by the effects of intracellular and extracellular current flows comprising sodium and chloride [4,28].

#### 6.2.4. Osmosis and Swelling of Cells

During a balanced equilibrium, factors such as cell volume and brain-cell environment are under a form of osmotic stability, which also maintains isotonicity [4,29]. Upon the onset of CSD, isotonicity no longer holds, instigating a redistribution of ions that subsequently allows water into the cells [4,29]. The osmotic pressure can be expressed as Equation (4):

(4) π=RTΨNc

where Ψ is the osmotic coefficient, which represents the degree of deviation from idealistic behavior; c is the molar concentration of the solute; and N is the number of ions that the compound breaks up into. If a solute exists on either side of the membrane, the net osmosis pressure provides a force to enable the migration of water through the cell. During the occurrence of CSD, the influx > efflux leads to a higher osmotic pressure in the extracellular space, which also drives water into the cell [4,29]. Thus, in the extracellular space, the rate of change of the fraction of the flow f to the flow of water across the membrane as a result of the osmotic pressure difference for a single ion can be represented as Equation (5):

(5) $$\frac{\partial f}{\partial t}=-\frac{P_{f}V_{w}S}{V}\left({\left[ion\right]}_{i}-{\left[ion\right]}_{e}\right)$$

where $f=\frac{V_{e}}{V}$, and $V_{e}$ represents the volume of the extracellular space, while $V_{i}$ is the volume of the intracellular space, and $V=V_{e}+V_{i}$ represents the total fixed volume, $P_{f}$ is the permeability of the osmotic water within the membrane, $V_{w}$ represents the partial molar volume, and S represents the surface area of the membrane. The osmotic pressure difference due to the ion recombination undergoes a modification of sorts, which yields the following relationship in Equation (6):

(6) $$\frac{\partial f}{\partial t}=-\frac{P_{f}V_{w}S}{V}\left(\;{\left[Na\right]}_{i}+{\left[K\right]}_{i}-{\left[Cl\right]}_{i}-{\left[Na\right]}_{e}-{\left[K\right]}_{e}+{\left[Cl\right]}_{e}+\frac{V_{i0}}{V_{i}}A\right)$$

In this equation, $V_{i0}$ represents the resting value of $V_{i}$ and $A={\left[Na\right]}_{e,0}+{\left[K\right]}_{e,0}-{\left[Cl\right]}_{e,0}-{\left[Na\right]}_{i,0}-{\left[K\right]}_{i,0}+{\left[Cl\right]}_{i,0}$ is the cumulative sum of the resting value of the ionic concentration, which ultimately yields the concentration of the immobile anions engrained within the cells.

## 7. Auxiliary Mechanisms

### 7.1. Glutamate

The work conducted by van Harreveld highlighted the role of glutamate in CSD, where the release of ATP from the astrocytes pulses independently and yields the release of glutamate from said astrocytes [4,30]. The effect of the glutamate acts on the N-methyl-D-aspartate to cue in a release of a considerable level of depolarization of the membrane potential, as well as a subsequent release of the glutamate, which forces further release of ATP to round up the phase [4,30]. (Although it is worth noting that there are separate theories around there being a duality between both glutamate and potassium in the process.)

### 7.2. Vascular Effects

It is well observed that CSD occurs with vascular changes that yield the manifestation of a migraine, where a two-phase neurovascular function has been recognized, which occurs during the pulsing of the CSD wave and is succeeded by a prolonged wave after the initial wave [4,31]. The by-product of this dynamic is a direct current shift alongside the constriction of the arteries as well as desaturation of the cortical hemoglobin.

Once the initial wave has concluded, a second wave follows, which is much more stretched out and prolonged and is characterized by a negative direct current shift, constriction of the arteries, and the desaturation of hemoglobin. This occurs alongside a continuous interference with the state of the neurovascular coupling, which leads to a lack of coherence and synchronism between the electrophysiological and perfusion acts.

It is also said that the occipital γ-aminobutyric acid (GABA), whose role is to reduce neuronal excitability by stopping nerve transmissions, is seemingly lower in patients with MWA when compared with patients without it.

## 8. Comparison of Mathematical Models for CSD

When it comes to the mathematical modeling of CSD, there are two key components to the accompanying mathematical models, i.e., the emanation of the CSD itself and the subsequent propagation of the CSD waves. The following represents a review and description of a shortlist of eight CSD models:

### 8.1. Tuckwell–Miura Model

Related studies have shown that the presence of tetrodotoxin within regions of the brain did not completely prevent the propagation of CSD and thus led to the inference belief that transient sodium membrane potentials could potentially be ignored from subsequent mathematical models of the phenomenon [4,32]. This led to the postulation by Tuckwell and Miura around neurotransmitters and increased extracellular site potassium. Due to this, presynaptic boutons will undergo depolarization, which would allow an influx of calcium and lead to an exocytosis of neurotransmitter molecules, which further opens up channels of potassium into the extracellular sites [4,32]. It is said that the novel insight mathematically offered by Tuckwell–Miura, was based around their mathematical description of brain activity, where due to the discrete and isolated veins of neurons, the propagation of CSD occurs in the order of minutes and thus enables the majority of the associated capacitive currents to be deemed negligible during the CSD waves [4,32]. This, along with the distinct point that the spatial scale of the waves of the CSD is much longer when compared to the size of the soma cell, implies that ionic concentrations both in the intracellular and extracellular space vary very little from one neighboring location to another [4,32]. The model proposed by Tuckwell and Miura assumes tissues to be a set of overlapping continuum spaces due to the extracellular and intracellular spaces hosted within the same space for both neurons and glia cells [4,32]. A key difference between intracellular and extracellular sites is the lack of diffusion in the case of the intracellular sites, where dynamic movement of neurons in the intracellular sites is first carried out by the crossing of the membrane in the extracellular site and then entering a neighboring neuron [4,32].

The use of continuum spaces with an associated overlap allows the use of differential equations without the need for interior boundaries; in this case, a continuum model in 1-dimension was adopted with only two ions in addition to potassium and calcium, which deal with the release of neurotransmissions [4,32]. Tuckwell and Miura’s models were seen to take into consideration diffusions of the extracellular potassium and calcium, as well as mechanisms at the membrane levels such as diffusion ions, ionic membrane currents, and electrogenic pumps, as described in Equations (7)–(10):

(7) $$\frac{\partial {\left[K\right]}_{e}}{\partial t}=D_{k}\frac{\partial^{2}{\left[K\right]}_{e}}{\partial x^{2}}+\rho_{1}\left(I_{k}+I_{k.pump}\right)$$

(8) $$\frac{\partial {\left[K\right]}_{i}}{\partial t}=\frac{\alpha }{1-\alpha }\rho_{1}\left(I_{k}+I_{k.pump}\right)$$

(9) $$\frac{\partial {\left[Ca\right]}_{e}}{\partial t}=D_{ca}\frac{\partial^{2}{\left[Ca\right]}_{e}}{\partial x^{2}}+\rho_{2}\left(I_{ca}+I_{ca.pump}\right)$$

(10) $$\frac{\partial {\left[Ca\right]}_{i}}{\partial t}=\frac{\alpha }{1-\alpha }+\rho_{2}\left(I_{ca}+I_{ca.pump}\right)$$

where ${\left[K\right]}_{e}$, ${\left[Ca\right]}_{e}$, ${\left[K\right]}_{i}$, and ${\left[Ca\right]}_{i}$ represent the ionic concentrations for potassium and calcium in the extracellular and intracellular sites, $D_{k}$ and $D_{ca}$ represent the coefficients for the ion diffusion for potassium and calcium, respectively, and $\rho_{1}$ and $\rho_{2}$ are constants that signify channel distributions and cell membrane area. $I_{k}$, $I_{k.pump}$, $I_{ca}$ and $I_{ca.pump}$ are all related to the membrane ionic currents represented in the above equation with dependencies of voltage and time conductances, as well as the energy-consuming pumps for both potassium and calcium, while α represents the fraction of the volume occupied by the extracellular space. Although it is acknowledged that there may exist a degree of fluctuation in the diffusion coefficients due to external factors such as temperature, etc., within the brain, the models presented here assume that these fluctuations are negligible, so constant values are adopted instead [4,32]. As part of the models, Hodgkin–Huxley voltage-dependent potassium conductance as well as voltage-dependent calcium conductance and pump expressions were chosen in order to reset the extracellular potassium and intracellular calcium to their baseline values [33].

### 8.2. Shapiro Model

The work conducted by Shapiro finely builds on the Tuckwell and Miura model with the adoption of a continuum model for the ionic concentrations of the extracellular and intracellular site overlaps [34,35]. Shapiro’s model was based around the inclusion of reaction-diffusion equations, which can characterize the flux variations of ions. In contrast to the Tuckwell–Miura model, which details reactions of the CSD on a cellular scale, Shapiro aimed to describe this on a more macro level [34,35]. In addition to this, two key inclusions are considered, i.e., first, the ionic diffusion in the intracellular sites via the neuronal junctions plays a pivotal role in the propagation of CSD, where—as a surrogate for the metrics of the gating property—a diffusion model was assembled, which is dependent upon the voltage variation across the gap junction in addition to the ionic concentrations [34,35]. And second, there is an assumption that the ionic concentrations from the CSD propagation will give rise to an osmotic effect that will lead to the swelling of cells.

Shapiro’s view and model also gave insights on ionic fluxes during CSD, with 20 different channels, pump currents, and concentrations of nine ion species, which include potassium, sodium, chloride, and calcium in both the extracellular and intracellular sites, with the ninth being buffered calcium [34,35]. All ionic concentrations are supplemented with Hodgkin–Huxley activations, which account for inositol triphosphate in the extracellular sites, contribute towards cellular signal transduction in the cells, and are assumed to be of importance in the activation of calcium channels within the endoplasmic reticulum, which serves as a calcium buffer [34,35].

The inositol triphosphate is said to be induced by an elasticated mechanism that is also degradable, but simulation results indicate that a diffusion act in the extracellular sites is not needed for the spread of the waves of the CSD [34,35]. Rather, the movement of ions through gap junctions allows for an alternate means of moving ions, so the blocking of gap junctions was viewed as a potential way of inhibiting the spread of the CSD waves [34,35]. Carbenoxolone, which is an active compound that is known for blocking junction gaps, still serves as an acceleration of the propagation of CSD and therein showcases that the blocking of gap junctions will not inhibit the spread of CSD itself [34,35].

The contribution of osmotic swellings and CSD was also looked at, and without considering the swelling of cells, it was seen that CSD waves would not propagate [34,35]. However, this feat and observation have varied based on the studies, where ignoring the effect of cell swelling in some cases has not completely inhibited the propagation of CSD waves [34,35].

### 8.3. Kager–Wadman–Somjen Model

As part of the work conducted by these authors, CSD membrane-based depolarizations with respect to a single neuron were investigated using the NEURON computational software, where a singular isolated neuron was immersed in an extracellular substrate to form a two-compartment system [4]. With this, both the intracellular and extracellular site ion concentrations were allowed to vary due to the presence of cross-membrane ion fluxes [4]. This model also included detailed information on the dendritic structures and the ion dynamic movements in various aspects of the dendrites due to concentration differences [4]. Simulation results showcased that the CSD-induced depolarization can be kickstarted either by a jolt of current or by nulling out the exchange of Na-K in the neuronal membrane [4]. The model was effectively comprised of a scale-down, with essential features being retained, but nevertheless, the model is slightly unreflective of the broad-picture manifestation of CSD, as the phenomenon cannot be robustly investigated with the use of a single neuronal model [4].

### 8.4. Bennett–Farnell–Gibson Model 

With the knowledge that glutamate is a key agent in the pulsing of the CSD waves alongside ATP, this proposed model comprised a 1-dimensional array of both astrocytes and neurons, which were intertwined via bidirectional communication, and facilitated by the NMDA receptors within the neuronal membranes and activated by glutamate [4]. The NMDA are accounted for in this model as glutamate-sensitive components that do not directly depend on potassium, as seen in Equation (11):

(11) $$I_{NMDA.ion}=P_{open}\frac{g_{ion.GHK}FE_{m}\left({\left[ion\right]}_{i}-exp\left(-\frac{E_{m}}{\emptyset }\right){\left[ion\right]}_{e}\right)}{\emptyset \left(1-\exp\left(-\frac{E_{m}}{\emptyset }\right)\right)}$$

Here, $\emptyset =\frac{RT}{F}$, where R and T represent the universal gas constant and absolute temperature, and F is the Faraday constant, $E_{m}$ is the membrane potential, ${\left[ion\right]}_{i}$ and ${\left[ion\right]}_{e}$ represent the ionic current intracellular and extracellular sites, $P_{open}$ is a glutamate-dependent channel probability expressed as $\frac{dP_{open}}{dt}=r_{1}{\left[Glu\right]}_{A}\left(1-P_{open}\right)-r_{2}P_{open}$, where $r_{1}$ and $r_{2}$ are both constants and ${\left[Glu\right]}_{A}$ is the concentrations of the glutamate released by astrocytes. The biochemical pathways, which involve both astrocytes and neurons undergoing ATP stimulation, are modeled in Equation (12):

(12) $$\frac{\partial {\left[Glu\right]}_{A}}{\partial t}=\frac{V_{A}{\left[ATP\right]}^{0.98}}{7+{\left[ATP\right]}^{0.98}}-\gamma_{A}{\left[Glu\right]}_{A}$$

where $V_{A}$ and $\gamma_{A}$ represent constants, and [ATP] is the local ATP released by astrocytes. The first and second terms on the right-hand side represent the production of glutamate and the loss of glutamate due to absorption, respectively. The concentration of glutamate released by neurons is identified by ${\left[Glu\right]}_{N}$, and is expressed as Equation (13):

(13) $$\frac{\partial {\left[Glu\right]}_{N}}{\partial t}=V_{N}e^{-0.0044{\left(E_{m}-8.66\right)}^{2}}-\gamma_{N}{\left[Glu\right]}_{N}$$

where $V_{N}$ and $\gamma_{N}$ represent constants. It can be noted that the release of glutamate from neurons hinges on the membrane potential, while that of astrocytes depends on ATP. IP3 serves as an agent that cues the release of ATP into the extracellular sites, where this compound is released from the astrocytes, followed by its concentration decaying due to degradation and spatial diffusions, as expressed in Equation (14) [4]:

(14) $$\frac{d{\left[IP_{3}\right]}_{j}}{dt}=r_{h}{\left(G_{ATP}^{*}+G_{Glu}^{*}\right)}_{j}-k_{deg}{\left[IP_{3}\right]}_{j}+\gamma \left({\left[IP_{3}\right]}_{j-1}-{\left[IP_{3}\right]}_{j}\right)$$

for which the concentrations are measured at the neighboring sites of the astrocytes (j − 1 and j), with $r_{h},\;k_{deg\;}and\;\gamma$ all representing constants. The fraction of G-protein that is ATP-activated is shown in Equation (15):

(15) $$G_{ATP}^{*}=\frac{\rho_{ATP}+\delta_{AT}}{K_{ATP}+\rho_{ATP}+\delta_{ATP}},\;\rho_{ATP}=\frac{\left[ATP\right]}{K_{RAT\;P}+\left[ATP\right]\;}\;$$

with $\delta_{ATP}$ being a constant. Further fractions of G-proteins can be cued in by glutamate signaling in the neurons, as seen in Equation (16):

(16) $$G_{Glu}^{*}=\frac{\rho_{Glu}}{K_{Glu}+\rho_{Glu}},\;\rho_{Glu}=\frac{\left[Glu\right]}{K_{RGlu}+{\left[Glu\right]}_{N}\;}$$

The extracellular potentials ($V_{SD}$) can be calculated using Equation (17):

(17) $$\frac{\partial V_{SD}}{\partial x}=-\frac{RT}{F}\underset{ion}{\sum }Z_{ion}D_{ion}\;\frac{\partial {\left[ion\right]}_{e}}{\partial x}{\left(\underset{ion}{\sum }z_{ion}D_{ion}{\left[ion\right]}_{e}\right)}^{-1}$$

where $Z_{ion}$ represents the valence of each ion as well as the extracellular concentration ${\left[ion\right]}_{e}$ that can be interpolated for.

### 8.5. Dahlem Model

In contrast to the prior described works, where contributors have mostly modeled individualistic characteristics of ions, neurons, etc., the model by Dahlem et al. is more poised around phenomenological mean field characterizations, which largely aim to separate theoretical preconceptions from directly observed manifestations themselves [4,36]. A dynamical systems perspective was adopted towards this characterization, from which it was deduced that CSD waves from migraines are not typical waves that pulse outward from the cortex, but rather, they are intermittent throughout the cortex, as has been observed in patients undergoing severe migraines, i.e., distinct and localized transient waves [4,36].

The work conducted by Dahlem et al. utilized reaction-diffusion models, which provided a form of generalization to the existing contributions by Hodgkin and Huxley, as expressed in Equations (18) and (19):

(18) $$\varepsilon \frac{\partial u}{\partial t}=u-\frac{u^{3}}{3}-v+\nabla^{2}u$$

(19) $$\frac{\partial v}{\partial t}=u+\beta +K\int H\left(u\right)dxdy$$

where u and v represent variables that serve as activators and inhibitors, respectively; H is the heavyside function; K is the mean field inhibitory coupling which represents vascular feedback; and β stands for a control-based parameter which indicates the dynamics of the model.

When K = 0, and there is no mean field coupling, the system is said to be bistable, while the homogeneous state, u = 0, represents a normal resting state, whereas a non-homogeneous state where u > 0 and related to the CSD is also viewed as stable. Once a critical mass is reached and the process of critical nucleation kicks in, CSD commences and permeates different neighboring regions upon commencement [4,36]. When K > 0, the critical mass process is regulated by an inhibitory feedback signal, which causes the traveling wave to be no longer global and, from there, local until attenuation continues to the point of disappearance. This manifestation is deemed saddle node bifurcation behavior, which effectively characterizes the propagation and dynamic pattern of the CSD waves in the presence of migraine [4,36].

### 8.6. Huang–Miura–Yao Model

This model poses a modified version of the Kager–Wadman–Somjen model, where the soma-dendrite system was replaced with a point neuron while also retaining the core characteristics of the cross-membrane currents for both sodium and potassium ions [34]. This is modeled as Equation (20) for the membrane potential:

(20) $$C_{m}\frac{dE_{m}}{dt}=-I$$

where t is time, $C_{m}$ represents the membrane capacitance per unit surface area, and I is the total cross-membrane ionic current per unit surface area. The cumulative current due to sodium ions is given as follows: $I_{Na}=I_{Na.T}+I_{Na.P}+I_{Na.Leak}+I_{Na.Pump}$, where $I_{Na.T}$ represents transient sodium currents as well as fast sodium currents, involved in action potentials; $I_{Na.P}$ is the persistent sodium current; $I_{Na.Leak}$ is the sodium leak current; and $I_{Na.Pump}$ represents the sodium exchange pump current tasked with restoring the ionic sodium concentration back towards homeostasis. The potassium current is also a factor in the total cross-membrane current and is expressed as $I_{K}=I_{K,DR}+I_{K.A}+I_{K.Leak}+I_{K.Pump}$, where $I_{K,DR}$ represents the delayed rectifier potassium current, $I_{K.A}$ is the potassium A current, $I_{K.Leak}$ represents the potassium leak current, and $I_{K.Pump}$ is the potassium exchange pump current. The dynamic time evolution of the concentrations of potassium and sodium ions is influenced by the cross-membrane currents as expressed in Equations (21) and (22):

(21) $$\frac{d{\left[ion\right]}_{t}}{dt}=-\frac{S}{FV_{i}}I_{ion}$$

(22) $$\frac{d{\left[ion\right]}_{e}}{dt}=-\frac{S}{FV_{e}}I_{ion}$$

where for the place of ion = Na, and K and S, $V_{i}$ and $V_{e}$ represent the surface area of the soma as well as both intracellular and extracellular volumes. Here, the spreading of the membrane depolarization is assembled using a series of point neurons, which are presumed to be 1-dimensional arrays with adequate spacing between them to mimic that of the brain [4,34]. The sodium and potassium ions move into the extracellular sites in the presence of diffusions, with the underpinning Equations (23) and (24) given as follows for a *j*th neuron, as well as the extracellular sites associated with it:

(23) $$\frac{d{\left[Na\right]}_{e,j}}{dt}=\frac{S}{FV_{e}}I_{Na,j}+\gamma_{Na}({\left[Na\right]}_{e,\;j+1}+{\left[Na\right]}_{e,\;j-1}-2{\left[Na\right]}_{e,j}$$

(24) $$\frac{d{\left[Na\right]}_{e,j}}{dt}=\frac{S}{FV_{e}}I_{K,j}+\gamma_{K}({\left[K\right]}_{e,\;j+1}+{\left[K\right]}_{e,\;j-1}-2{\left[Na\right]}_{e,j}$$

where $\gamma_{Na}$ and $\gamma_{K}$ represent coefficients from molecular diffusions, $D_{Na}$ and $D_{K}$, which are scaled by the square of their average distance between the neurons represented by $\left(\delta \right)$, i.e., $\gamma_{ion}=\frac{D_{ion}}{\delta^{2}}$.

For an application of an electrical stimulus for a timespan of around 200 ms to an arbitrary number of three neurons, a depolarization effect occurs upon a prolonged timespan of action potentials [4,34]. This cues in a domino effect of the extracellular site potassium concentration and a subsequent influx of sodium ions. Due to the knock-on effect of this, action potentials become induced in neighboring neurons and ultimately result in a series of depolarizations of the neuronal membrane potentials [4,34]. This simplified network of neurons has been used as an exploratory basis for the membrane-spreading depolarization caused by the application of KCl in the absence of Na. Here, KCl is assumed to be mainly confined to the extracellular sites of the first three neurons, which are almost instantaneously depolarized with KCl, while the subsequent neurons go through the depolarization one after the other without any direct firing of action potentials [4,34].

Their models were also extended towards an observation of the role of NMDA currents, which occur mainly in dendrites, by the inclusion of another compartment. From this, it was noted that upon the emanation of a CSD wave, its propagation dynamics are in a similar vein to ordinary waves that occur in the absence of dendrites. However, the blocking of the NMDA channels was noted to be able to prevent the CSD wave from starting and spreading due to the membrane potentials in the neuronal soma being substantially modified [4,34]. Also, if the NMDA channels are blocked off in the dendrites of neurons away from the region where the KCl is induced, it is noted that the CSD waves are not completely blocked off at this point [4,34].

This model also enabled quantitative predictions to be made through auxiliary observations quantifying the speed of the propagation of the CSD waves. It is hereby mentioned that discrete models can be unfavorable due to their computational burden, thus promoting the application of continuum models.

### 8.7. Yao–Huang–Miura Model

This model allows for intracellular and extracellular volume changes; it does not make assumptions in terms of neurotransmitters and features a more robust repertoire of membrane ionic currents [4]. Here, the concentration of sodium in both the intracellular and extracellular volume changes is governed by the following Equations (25) and (26):

(25) $$\frac{\partial {\left[Na\right]}_{e}}{\partial t}=\frac{S}{FVF^{1}}I_{Na}-\frac{{\left[Na\right]}_{e}}{F^{1}}\frac{\partial F^{1}}{\partial t}+\frac{1}{F^{1}}\frac{\partial }{\partial x}\left(F^{1}D_{Na}\frac{\partial {\left[Na\right]}_{e}}{\partial x}\right)$$

(26) $$\frac{\partial {\left[Na\right]}_{i}}{\partial t}=\frac{S}{FV\left(1-F^{1}\right)}I_{Na}+\frac{{\left[Na\right]}_{i}}{1-F^{1}}\frac{\partial F^{1}}{\partial t}$$

where $F^{1}=V_{e}/V$, while the first terms to the right of both equations represent the membrane current of sodium, the second is for the extracellular and intracellular volume changes, and the third and final set of terms represent the diffusive flux of sodium in the extracellular site [4]. This model was supported by numerical simulations by their inceptors to observe the dynamics of the cortical spreading waves [4]. When the cell volume and concentrations were constant, the CSD waves intensified in synchronism with decreasing $F^{1}$, which was seen to be consistent with an expanded extracellular space and ultimately cause a slower buildup of the extracellular potassium concentration [4].

### 8.8. Neurovascular Coupling Model 

None of the reviewed models above have included the features and connections that the vascular system imposes on the supply of oxygen or the auxiliary neurochemicals that the brain is dependent upon during the wave of CSD [4,21]. Part of this model is a sodium-potassium-ATPase that is chiefly responsible for ionic homeostasis and is active during CSD at a rate that is dependent on the flow of oxygen, while the supply of oxygen itself is determined by a lumped vascular tree identification with a local vessel radius that is regulated by an extracellular potassium concentration [4,21].

Both the qualitative and quantitative dynamics of the CSD were included as part of this model, i.e., vasoconstrictions, oxygen, potassium elevation, etc., which were noted from experimental studies and showcase the aftereffect of the scarcity of oxygen on the CSD recovery.

It has been previously noted that the metabolic demands apparently exceed physiological limits placed on the generic delivery of oxygen irrespective of the constriction or dilation of the vasculature (although it also needs to be mentioned that both constriction and dilation of the vasculature are pivotal in the proneness of the cortical tissue to CSD and its recovery) [4,21]. This case study model was also able to incorporate both perfusion and metabolic factors into its model to help further explain the phenomenon both in vitro and in vivo.

A summary of the various kinds of models, along with their key features and characteristics, can be seen in Table 1.

## 9. Linking Known Triggers to Aura Manifestation

### 9.1. Food

Due to the chemical properties of food, various kinds have been reported as potential triggers for MwA, where red wine has been identified as a potential trigger due to its constituents leading to an interplay of sorts between ions and neurotransmitters [37,38]. Consumption of chocolate has been identified as a trigger by around 20% of the participants of a survey. While it is known that chocolate affects brain oscillations, in particular increasing alpha and beta activities whilst also dropping the theta and delta activities, there continues to be research conducted around further investigating the direct link of chocolate as a trigger [37,38].

### 9.2. Sensory Factors

Recent research has found that migraine-based sensory triggers are much more unique and distinct from typical headache triggers, where common optical and visual stimuli include bright light, reading, striped patterns, and sharp colors, while light has also been reported as a strong trigger in a particular younger cohort of 10–19 years [37]. This study has also shown that flickering lights have been seen to be a source of migraine triggers, where 5 Hz flickering stimulations were utilized and a variety of exposure times led to migraines [37]. It was also seen that the maximum exposure time did not always lead to the most intense headaches, which is indicative that habituation to the stimuli also plays a contributing factor [37].

### 9.3. Auxiliary Factors

As detailed, the majority of migraine triggers vary between the various cohorts, which renders them challenging to succinctly pinpoint [9]. However, some triggers can be effectively “reverse engineered” and give an idea of brain states during their manifestation, i.e., stress and sleep deprivation, which can be correlated to the presence (or lack) of potassium, while lowering the level of glutamine through repetitive light stimulation has been seen to have some effect on people with migraines for whom the baseline glutamine levels were elevated [9].

## 10. Correlations between Theoretical Knowledge and Physiological Measurement Instrumentations

Brain recordings have been utilized in different capacities to observe if theoretical postulates and assumptions can be linked with data-driven observations from physiological measurement modalities, i.e., EEG and fMRI. Although scalp EEGs have been mentioned to be too superficial to adequately track MwA manifestations, nevertheless, abnormal EEGs have been noted in individuals with MwA, where these EEGs are characterized by slow dynamic waves and spiking activities [9]. The research assembled for the “Brain Engagement Index” has shown correlations with MwA patients, where there has been seen to be a pre-ictal stage (where ictal refers to the attack stage) that has been succeeded by a saturated sequence afterwards [9].

Although interictal EEG measurements during MwA have been challenging to come across, there have been a handful of instances where data has been assembled and qualitatively observed in patients with frequent chronic neurological attacks.

### 10.1. Slow Wave Oscillations

Slow wave oscillations in the region of 5–8 Hz have been previously reported with participants experiencing MwA-type headaches, while also in basilar-type migraine (BAM), whose neural manifestation is similar to MwA, slow wave activities have been mentioned during the attacks in the posterior section of the brain, albeit mostly the theta bands in a diffuse and continuous pattern. PET scans and acquisitions of patients with spontaneous auras showcased showed a reduction in blood flow over time described as hyperfusions [9], manifestations of which are said to link neuronal excitability, spreading depolarizations, and depressions with electrophysiological activities [9].

### 10.2. Electrophysiology and Reaction-Diffusion Oscillations

Despite the alpha band being seen as an idling rhythm within the brain, alpha band oscillations have been viewed as a “window of excitability”, for which when a designated flicker is synchronized with an individual’s alpha band oscillations, EEG recordings have been able to show that traveling waves of around 5 s worth of emergence are relative to 15 s worth of trials in the non-synchronized states [39,40,41]. These traveling waves eventually morph into hallucinatory patterns, which can occur even in an eye-closed situation [39,40,41]. Hallucinatory patterns can be typically modeled using reaction-diffusion models alongside energy models, which show the dissipation and dynamic energy of the wave motions and are key for showing the relationship between a source input frequency and an accompanying hallucination [39,40,41].

### 10.3. Electrophysiology and Brain Connectivity

It needs to be stressed and ascertained that brain recordings using different kinds of measurements, i.e., EEG and fMRI, do not represent the same scale of information as derived mathematical models due to these being on a cellular scale, whereas the likes of the aforementioned physiological measurements represent information on a different scale due to their spatial resolutions [42,43]. Nevertheless, the MwA is notedly characterized by distinct oscillatory differences that involve slow-wave activity, a reduction in alpha power, and increased beta power. Key factors that are assumed to cue migraines include the deprivation of sleep and photic simulations, which can cause changes in EEG recordings [42,43].

## 11. Future Research Pathway

- There continue to be uncertainties around how CSD accounts for complex auras and hallucinations; for example, bilateral visual auras, which are observed in various hemispheres, are not accounted for by CSD since their wave propagations require contiguous gray matter and do not impede across the corpus callosum [9]. As the spreading depression mostly propagates in the region and hemisphere of origin, it does not fully account for the bilateral pain, which 40% of people with migraines experience [9]. Hence, it needs to be acknowledged that most models are based on trivial geometric hallucinations;
- More complex hallucinations also occur, with complexities that go beyond the typical flashing pattern. These complex illusionary patterns are not incorporated in the majority of current models due to their initiation stemming from an unaccounted secondary source [9];
- Migraines, although largely formulaic in their manifestations, are unique in their dissipations within the brains of various individuals; thus, current models are not robust enough to take into account individual variabilities [9]. Thus, there are ploys towards amalgamating data-driven observations from MRIs and EEGs towards seeking means to account for and model individual distinct differences during CSD and MwA [9];
- Continued questions linger around the degree to which models reflect individual susceptibilities to MwAs, where alpha bands have been shown to be indicative of spreadings as well as “internal noise” ratios, which can be assessed with model-based parameters [9];
- There are uncertainties around how the established models (described in Section 8) effectively account for patient pain and discomfort due to cases of painless MwA, for which simulated case studies showed that the spreading depression waves arrived at the primary and secondary somatosensory cortex, including the area linked to the trigeminal nerve as well as the pain processing prefrontal areas [9]. This implies that the pulsing of the extracellular K+ would cue in the headache by initiating the pain receptors, therein providing evidence that the depression spreading process incorporates neuronal activation protocols that are tasked with mediating lateral head pain [9]. Subsequent work on this theme would look towards linking spreading depressions and nominal head pain, using Laplacian functions and reaction-diffusion models as a basis;
- There are concerns about the long-term impact and effects of continued migraine attacks, which have been seen in certain cases to progress into chronic migraine syndrome, resulting from a heightened level of sensitization due to a repeated streak of depolarization and depression mechanisms [44]. Despite the benign nature of migraines, there are mentions of possible effects such as an increased risk of stroke, which is a disorder resulting from spreading depolarization [44]. Thus, the risk and likelihood of this are ones that need to be explored further [44];
- The bulk of the work offered as part of this contribution is themed around models and observations made from an adult population that is largely believed to be male; thus, more targeted research in their female counterparts and in the pediatric migraine cohort needs to be conducted to help bolster up models to be more robust for age and sex-based factors [9]. Furthermore, hormonal differences are said to be the reason behind an increased occurrence of migraines in females when compared to males, where MwA attacks are thought to be due to a high level of estrogen that occurs in pregnancy, for example. Links between estrogen peaks and MwA are thought to be associated with an increment in excitability in the visual cortex [9];
- In order to further closely monitor patients who suffer MwA attacks, a combination of mobile health monitoring platforms could be adopted due to the intermittent nature of the attacks. This would involve a set of wearable brain monitors combined with signal processing and machine learning analytics, which could allow for an almost real-time update on the brain state before, during, and after migraine attacks [45,46].

## 12. Concluding Remarks

The use of mathematical models within clinical medicine is advantageous in helping to gain a deeper understanding of the manifestations themselves as well as serving as a mechanism towards understanding numerous what-ifs in various scenarios in a controlled and safe manner [47,48,49,50]. This work has surveyed some key primary models, as well as their assumptions, for a robust understanding of the propagation dynamics of the CSD waves. The eight models surveyed showcase various aspects and segments of the manifestations based on the assumptions baked into them and the perspectives from which their simulations have been conducted. Although these models and their assumptions cannot thoroughly explain the CSD phenomenon, they do contribute towards a finer understanding of a certain occurrence, which in this case is also linked to the workings of the brain.

The observations from this exploratory review further showcase mathematically how various dynamical-based acts such as continuums, reaction diffusions, and single-point neurons can be accounted for and identified using mathematical models and formulae [51]. These mathematical tools and approaches can be extended towards numerical identifications and simulations of various kinds of psychiatric manifestations that require further study.

As part of the qualitative aspects surrounding migraines, factors such as migraine triggers have been explored with insights on what physiological mechanism triggers their onsets; links between the theoretical models and acquired physiological measurements have been discussed, as well as the link between electrophysiology and other brain behaviors [9]. Further work pathways accounting for individual variabilities, sex, age group, and hormonal factors pertaining to MWA have also been addressed.

## Figures and Tables

**Table 1:** Summary of the eight CSD models with their key features and characteristics.

Model	Features and Characteristics
Tuckwell–Miura	- Mathematical description of brain behavior with respect to discrete and isolated neurons, to model CSD effects
Shapiro	- An incremental advancement on the Tuckwell-Miura model, but this time with the use of continuum models for intra and extracellular site overlaps - Inclusion of reaction-diffusion models to characterize flux variations, with macroscale descriptions
Kager–Wadman–Somjen	- A single neuronal component for the observation of CSD manifestation on a deep scale perspective
Bennett–Farnell–Gibson	- Emphasized on the observation of the role of glutamate within the overall process
Dahlem	- Based around phenomenological mean field manifestations, which are aimed at distinguishing experimental manifestations from theoretical postulates
Huang–Miura–Yao	- Modified variant of the Kager-Wadman-Somjen model with a point neuron, but retaining core characteristics
Yao–Huang–Miura	- Makes provisions for intra and extracellular volume changes and a robust repertoire of membrane ionic currents.
Neurovascular coupling	- Factors in the link between the vascular system and neurochemicals within the brain

## Data Availability

Not applicable.
